# Evaluation of robotic exposure among gynecological surgeons: results of survey from the young European advocates of robotic surgery (YEARS)

**DOI:** 10.1007/s11701-026-03153-7

**Published:** 2026-02-11

**Authors:** Sergi Fernandez-Gonzalez, D. El-Hamamsy, E. Karatrasoglou, A. Amirthanayagam, A. Muñoz Solano, C. Chollet, D. Galvin, M. Manpreet Kaur, C. Uwins

**Affiliations:** 1 Department of Gynaecology, Bellvitge University Hospital (IDIBELL), L’Hospitalet de Llobregat, Spain; 2 Royal London Hospital, London, UK; 3 Euroclinic Group of Hospitals Athens, Alexandra University Hospital, Athens, Greece; 4 Leicester Cancer Research Centre, University of Leicester, Leicester, UK; 5 Hospital Universitario Marqués de Valdecilla, Santander, Spain; 6 Centre Hospitalier Universitaire de Toulouse, Toulouse, France; 7 Surgical Oncology, Institut Universitaire du Cancer Toulouse Oncopole, Toulouse, France; 8 Cork University Maternity Hospital, Cork, Ireland; 9 Chelsea and Westminster Hospital London, London, UK; 10 University Hospital Southampton NHS Foundation Trust, Southampton, UK

**Keywords:** Robotic surgery, Gynecology, Young surgeons, Surgical confidence

## Abstract

Study objective: To identify factors associated with higher self-perceived confidence in performing robotic surgery among early-career gynecologic surgeons, and to assess satisfaction with current surgical activity among young consultants. A cross-sectional survey consisting of a 28-item online questionnaire was developed and disseminated through the social media channels of the Young European Advocates of Robotic Surgery (YEARS) and the Society of European Robotic Gynaecological Surgery (SERGS). Eligible participants included gynecologists with exposure to robotic surgery who were either (1) still in specialty training or (2] within three years of completing their postgraduate training or fellowship. The questionnaire collected sociodemographic information, details on surgical experience, access to robotic platforms, and self-perceived confidence in different surgical approaches. Main results: A total of 81 respondents completed the survey. Among the 63 surgeons with access to robotic surgery at their institutions, 29 reported high self-perceived confidence and 36 reported lower confidence. Factors associated with higher confidence included age > 35 years (51.8% vs. 33.3%; *p* < 0.001) and having received structured robotic training through a fellowship or industry-sponsored course (40.7% vs. 19.4%; *p* = 0.017). Departments performing ≥ 55 robotic procedures per year showed a greater likelihood of hosting surgeons with high confidence. Finally, age > 35 years and access to at least one dedicated robotic operating day per week weres independently associated with higher confidence. Conclusion: Self-perceived confidence in robotic surgery among early-career gynecologic surgeons appears to be associated with age, and regular access to robotic operating time. Summation: Being older than 35 years and performing robotic surgery at least once weekly are associated with greater confidence in the robotic approach, emphasizing the importance of experience and exposure.

**Supplementary Information:**

The online version contains supplementary material available at https://doi.org/10.1007/s11701-026-03153-7.

## Introduction

 Gynecologic surgery has undergone a substantial technological evolution in recent decades, largely driven by the increasing global adoption of robotic platforms. More than two decades have passed since the first reported robotic-assisted hysterectomy in 2002 [1]. Since then, the use of robotic surgery has expanded steadily across both high-income [2] and low- and middle-income countries [3]. This trend contrasts with a documented decline in overall gynecologic surgical volume. For example, the incidence of hysterectomy in Denmark decreased from 362.9 per 100,000 person-years in 2000 to 307.1 in 2015 [4] while hysterectomy prevalence in the United States has remained stable or slightly decreased over recent years [5].

This discrepancy of growing implementation of high-technology surgical platforms and reduced operative volume, has raised concerns regarding the adequacy of surgical training for young gynecologists. Recent studies have reported reduced exposure to core gynecologic procedures during training. A national Irish survey of gynecology trainees showed a significant decline in confidence to perform abdominal and vaginal hysterectomies when comparing cohorts from 2014 to 2021 [6]. Likewise, data from the Australian Institute of Health and Welfare showed a reduction in major gynecologic procedures performed between 2013 and 2018, including laparotomies, vaginal hysterectomies, and continence surgeries [7]. Trainees performed fewer than 12 major gynecologic procedures per year on average raising concerns about potential implications for surgical safety and outcomes [8].

Despite the rapid expansion of robotic technology, there is limited published data describing the level of exposure to robotic surgery and the availability of structured training opportunities for early-career gynecologic surgeons in Europe. Understanding these patterns and their influence in self-perceived confidence may indicate how prepared surgeons feel and may help reveal gaps in training. Identifying which factors are associated with higher confidence can therefore provide insight into potential inequalities in access, surgical volume, and training pathways that influence a surgeon’s readiness to perform robotic procedures independently. In this context, the Young European Advocates of Robotic Surgery (YEARS) conducted a survey among early-career gynecologic surgeons across Europe to evaluate exposure to robotic surgery. The primary objective of this study was to identify factors associated with higher self-perceived confidence in performing robotic surgery. A secondary aim was to assess satisfaction with current surgical activity among young consultants.

## Methods

### Study design and participants

The YEARS Council developed a 28-item questionnaire following three rounds of expert consensus (Document S1). The target population consisted of early-career gynecologic surgeons with exposure to robotic surgery. Three inclusion criteria were defined for survey participation: [1] trainee/resident enrolled in a national obstetrics and gynecology training program; [2] gynecology clinical fellow; and [3] recent consultant (within the past 3 years) of a fellowship or sub-specialty training program.

### Survey structure

The questionnaire collected sociodemographic data, information on surgical experience, access to robotic platforms, and exposure to different surgical approaches including questions about gynecologic surgical training and the surgical activity of both the respondent’s department and the individual surgeon. It also assessed satisfaction with current surgical activity and self-perceived confidence in performing robotic procedures. Most items used predefined response categories, although free-text fields were included where appropriate. This approach ensured respondent autonomy and improved data completeness. Once the survey was completed and submitted, responses were automatically stored in the repository. Respondents were not able to review or change their answers after submission. To avoid duplicate entries, responses were cross-checked for internal consistency and stored in a confidential repository accessible only to the study investigators.

### Recruitment and data collection

The survey was hosted on SurveyMonkey and distributed through the social media accounts of YEARS and the Society of European Robotic Gynaecological Surgery (SERGS) inviting members to complete the 5-minute survey. When the survey was launched, the YEARS group had 65 registered members. Responses were accepted from December 2022 to September 2023, and all data were handled confidentially. This study was exempt from Institutional Review Board (IRB) approval as it involved an anonymous survey without collection of identifiable personal or clinical data. Preliminary results were shared in the annual SERGS conference in June 2023. Additionally, there was completed the Checklist for Reporting Results of Internet E-Surveys (CHERRIES), adhering to current methodological recommendations for the reporting of survey-based research (Document S2).

### Definition of confidence groups

Among respondents working in departments with robotic activity, two confidence groups were defined:

- The High-Confidence Group: included surgeons who identified robotic surgery as their approach associated with greater confidence and reported operating independently or with only minimal supervision.
- The Low-Confidence Group: included surgeons who selected another surgical approach linked to higher confidence or who required consultant assistance or supervision when performing robotic procedures.

### Statistical analysis

Descriptive and comparative statistics were applied to summarize the survey results. Student’s *t*-test and Chi-square (*χ²*) test were used to assess differences between continuous and categorical variables, respectively. The number of cases and percentages were presented for categorical variables while continuous variables are reported as median with range or mean with standard deviation (SD), depending on data distribution. Univariate and multivariate logistic regression models were performed to identify factors associated with higher surgical confidence. Odds ratios (OR) with 95% confidence intervals were reported. Statistical significance was set at *p* < 0.05. Additionally, a Receiver Operating Characteristic (ROC) curve was used to determine the optimal threshold for annual departmental robotic case volume associated with higher surgeon confidence. All analyses were conducted using SPSS version 25.0 (IBM Corp., Armonk, NY, 2018).

## Results

### Participant characteristics

A total of 81 respondents completed the survey. Most participants (65.4%, *n* = 53/81) were between 30 and 39 years of age, and the majority (70.4%, *n* = 57/81) reported a primary interest in gynecologic oncology (Table 1). Of all respondents, 53.1% (*n* = 43/81) were already practicing as consultants, while the remainder were still enrolled in a structured or unstructured training program. Among consultants, 9.3% (4/43) reported working in a different sub-specialty than the one they were trained in. Regarding robotic surgery training, 17.3% (*n* = 14/81) had not received any formal education in robotic surgery, and 22.2% (*n* = 18/81) perceived access to robotic training as difficult. Notably, robotic courses and webinars were the two preferred modalities for learning in 42% (*n* = 34/81) and 49.4% (*n* = 40/81) respectively. Finally, 80.3% (*n* = 65/81) of respondents expressed strong interest in undertaking a robotic surgery observership.

**Table 1:** Characteristics of respondents

	All (N=81)
*Respondent demographics and professional background*	
Age, years (%)	
<30	8 (9.9%)
30–34	27 (33.3%)
35–39	26 (33.1%)
≥40	20 (24.6%)
Gender (%)	
Female	44 (54.3%)
Male	37 (45.7%)
Level of experience (%)	
Currently, doing training program in gyn/obs	16 (19.8%)
Currently, 1/2 half of fellowship	12 (14.8%)
Currently, 2/2 half of fellowship	5 (6.2%)
Working as a consultant < 4 years	43 (53.1%)
Currently working in other competencies	4/43 (9.3%)
Others	5 (6.2%)
Area of interest (%)	
Gynecology Oncology	57 (70.4%)
Benign gynecology	18 (22.2%)
Urogynecology	6 (7.4%)
Subspecialty training program (%)	
Structured fellowship	33 (40.7%)
Non-structured fellowship	25 (30.9%)
Not enrolled in any subspecialty training program	21 (25.9%)
NA	2 (2.5%)
*Learning resources for robotic surgery*	
Robotic training (%)	
Fellowship	19 (23.5%)
Local hospital course	11 (13.6%)
Industry course	23 (28.4%)
National or international society course	14 (17.3%)
None	14 (17.3%)
What learning tool do you usually access regarding robotics, (multiple choice question, %)	
Dedicated robotic on-site courses	34 (42%)
Webinars	40 (49.4%)
Society websites	27 (33.3%)
Social media	12 (14.8%)
Would you like to do a short observerships dedicated to robotic surgery?	
Extremely interested	37 (45.7%)
Very interested	28 (34.6%)
Low interested	15 (18.5%)
NA	1 (1.2%)
How is access to robotic learning tools in your opinion?	
Easy access	20 (24.7%)
Moderate access, limited information	42 (51.9%)
Difficult access	18 (22.2%)
NA	1 (1.2%)

### Respondents’ experience with robotic surgery

Among the 81 respondents, 63 reported having robotic surgery available at their department and were included in the confidence analysis. Of these, 36.5% (23/63) had access to at least one dedicated robotic operating day per week. In contrast, 83.9% (52/62) indicated that their department performed robotic surgery at least weekly (Table 2). Only 17.7% (*n* = 11/62) of surgeons reported being satisfied with their current frequency of exposure to robotic surgery. Overall, 46% (*n* = 29/63) of respondents selected robotic surgery as their preferred surgical approach.

**Table 2:** Surgical activity among young consultants or fellows with access to robotic platform

	All (63)
Frequency of robotic surgery per department (%)	
< 1 day per week	10 (15.9%)
1 day per week	21 (33.3%)
> 1 day per week	31 (49.3%)
NA	1 (1.5%)
Robotic research per department (%)	
Yes, prospective	33 (54%)
Yes, retrospective	6 (9.5%)
No	23 (36.5%)
Robotic platform (%)	
Da Vinci Si	9 (14.3%)
Da Vinci X	11 (17.4%)
Da Vinci Xi	49 (77.8%)
Hugo	4 (6.3%)
Frequency of robotic surgery per young surgeon (%)	
< 1 day per week	39 (61.9%)
1 day per week	15 (23.8%)
> 1 day per week	8 (12.8%)
NA	1 (1.5%)
Level of confidence as a main surgeon by any approach (%)	
High confidence: no need of supervision	26 (41.3%)
Medium confidence: Assisted by fellows with some supervision	12 (19%)
Low confidence: Assisted by a consultant	20 (31.7%)
Low confidence: not ready as a main surgeon	5 (7.9%)
Surgical approach of preference (multiple choice question, %)	
Open	21 (33.3%)
Laparoscopy	23 (36.5%)
Robotics	29 (46%)
Not specified	10 (15.9%)
Satisfaction regarding frequency as a main surgeon by robotics	
No, I am happy with my frequency	11 (17.5%)
I would like to operate more often as a main surgeon	35 (55.6%)
I would like to initiate surgeries as a main surgeon	16 (25.4%)
NA	1 (1.5%)

**Fig. 1:**
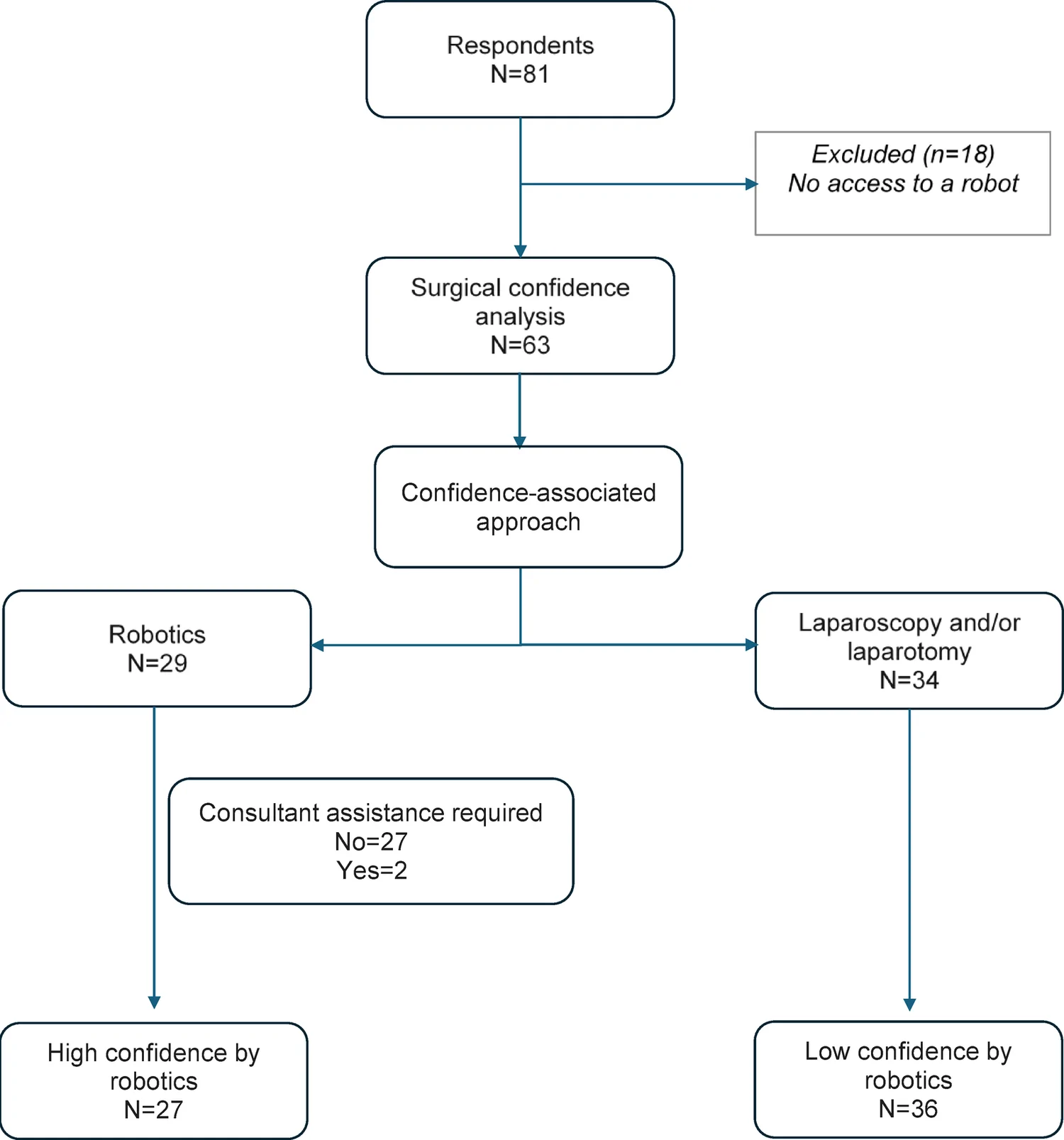
Flowchart

Surgeons who selected robotic surgery as their preferred approach and require “no” or “minimal supervision” when operating were classified into the High-Confidence Group (*n* = 29), while those who preferred another approach or required consultant assistance were classified into the Low-Confidence Group (*n* = 36) (Fig. 1).

Significant factors associated with high surgical confidence are shown in Table 3 and included being older than 35 years: 51.8% in the High-Confidence group vs. 33.3% in the Low-Confidence group (*p* < 0.001), and having received robotic training during a fellowship or industry-sponsored course in 40.7% vs. 19.4% respectively, (*p* = 0.017).

**Table 3:** Comparison between high-confidence group vs low-confidence group operating by robotics. Univariate analysis

	High confidence (27)	Low confidence (36)	*p value*
Age (years, %)			*<0.001*
< 35 years	4 (14.8%)	23 (63.9%)	
35–39 years	14 (51.8%)	12 (33.3%)	
≥ 40	9 (33.4%)	1 (2.8)	
Place of work			*0.748*
Tertiary University Hospital	21 (77.8)	26 (72.2)	
Tertiary No-University Center	3 (11.1)	6 (16.7)	
Private Hospital	3 (11.1)	4 (11.1)	
Area of interest (%)			*0.955*
Gyn-Oncology	20 (74.1)	27 (75)	
Benign gynecology (endometriosis, fibroids…)	5 (18.5)	7 (19.4)	
Uro-Gynecology	2 (7.4)	2 (5.6)	
Subspecialty training program			*0.680*
Structured fellowship	12 (44.4)	13 (38.2)	
Non-structured fellowship	9 (33.3)	10 (29.4)	
Not enrolled in any training program	6 (22.2)	11 (32.4)	
Robotic training			*0.017*
During my fellowship	11 (40.7)	7 (19.4)	
Industry course	11 (40.7)	7 (19.4)	
National/International society course	2 (7.4)	5 (13.9)	
Local hospital course	2 (7.4)	8 (22.2)	
Robotic research per department?			*0.462*
Yes, prospective	17 (63)	17 (47.2)	
Yes, retrospective	2 (7.4)	4 (11.1)	
No	8 (29.6)	15 (41.7)	
Frequency of robotic surgery per department (%)			*0.214*
> 1 day per week	16 (59.3)	15 (41.7)	
1 day per week	9 (33.3)	12 (34.3)	
< 1 day per week	2 (7.4)	8 (22.9)	
Robotic surgeries per year per department (median)	100 (10–450)	50 (10–300)	*0.05*
Frequency as main surgeon by robotics			*<0.001*
> 1 day per week	6 (22.2)	2 (5.6)	
1 day per week	13 (48.1)	2 (5.6)	
< 1 day per week	8 (29.6)	31 (88.6)	

**Fig. 2:**
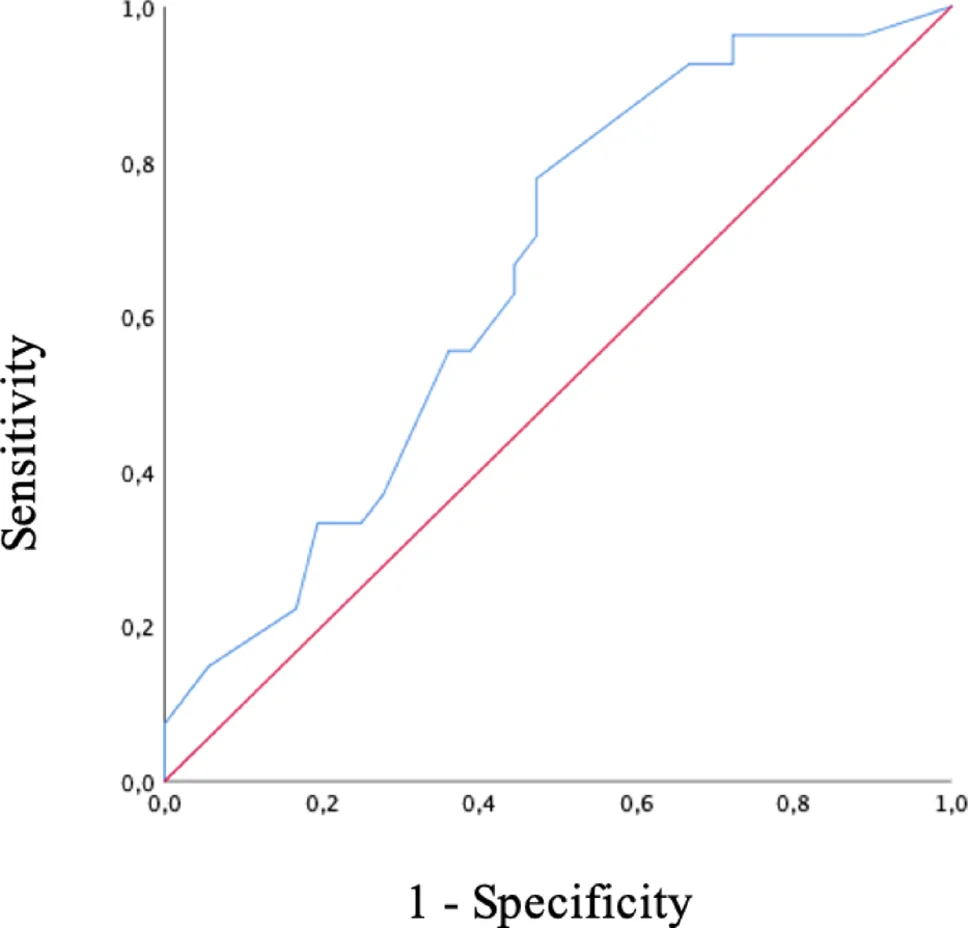
ROC curve for number of robotic surgeries per department per year

Departments hosting high-confidence surgeons also demonstrated higher median annual robotic surgical volumes, 100 vs. 50 cases, (*p* = 0.05). A ROC curve analysis identified 55 robotic procedures per year as the optimal threshold associated with higher levels of self-perceived confidence (AUC 0.655 [0.521–0.790], *p* = 0.036; Fig. 2). Notably, only 29.6% of surgeons in the high-confidence group operated robotically less than once per week, compared with 88.6% in the low-confidence group (*p* < 0.001).

The AUC for number of robotic surgeries per year is 0.655 (0.521–0.79) p 0.036.

55 robotic surgeries per year has 77% of sensitivity 77% and 47% of specificity.

In the multivariate logistic regression model (Table 4), age > 35 years and having access to at least one dedicated robotic operating day per week were independently associated with high surgical confidence.

**Table 4:** Independent factors for high confidence to operate by robotics. Multivariate analysis

	OR	95% Confidence Interval	*p value*
Age (years, %)			
< 35 years	Ref.		
≥ 35 years	0.116	0.025–0.533	*0.006*
Frequency as a main surgeon by robotics			
< 1 day per week	Ref.		
≥ 1 day per week	0.06	0.013–0.265	*<0.001*
Robotic training			
Fellowship	Ref.		
Industry course	0.142	0.009–2.208.009.208	*0.163*
National/International society course	0.306	0.043–2.167.043.167	*0.236*
Local hospital course	0.166	0.005–5.644.005.644	*0.319*

## Discussion

To our knowledge, no previous studies have specifically evaluated the factors associated with self-perceived confidence in robotic surgery among early-career gynecologic surgeons. In this survey, higher confidence was associated with being older than 35 years and with regular exposure to robotic procedures, particularly access to at least one dedicated robotic operating day per week. These findings suggest that both cumulative surgical experience and consistent operative practice may contribute to increased confidence during the early stages of a surgeon’s career. Importantly, these associations should be interpreted as correlations rather than evidence of causality.

Our results are consistent with previous studies evaluating preparedness in minimally invasive gynecologic surgery (MIGS). Trainees exposed to fellowship-trained MIGS surgeons report greater readiness for independent practice compared with those without such exposure [9]. Other studies have shown that fellowship-trained MIGS surgeons achieve improved surgical outcomes compared with gynecologic oncologists or general gynecologists performing minimally invasive hysterectomies [10, 11]. Conversely, a retrospective analysis of the impact of urogynecology fellowships on obstetrics and gynecology (OB/GYN) residency programs found that graduates from fellowship-affiliated institutions logged fewer cases overall compared with programs without such fellowships [12]. Taken together, these findings highlight the complex relationship between training structure, surgical volume, and procedural autonomy [7].

In our cohort, departments performing at least 55 robotic procedures per year were more likely to host surgeons reporting higher confidence. Although this threshold should be interpreted cautiously, it may reflect the minimum case volume required to provide trainees with a regular opportunity to participate in robotic surgery. Multiple observational studies have shown that proficiency is typically achieved after approximately 20–50 cases [13–15], depending on surgeon experience, case complexity, and robotic platform. In addition, Platform-specific analyses further highlight the importance of cumulative exposure. Vizza et al. [16] demonstrated that surgeons transitioning from multiport to single-port robotics achieved mastery after only 13 cases, whereas approximately 50 cases were required to reach mastery with the multiport platform. These thresholds align closely with our ROC-derived cut-off of 55 cases per year, suggesting that departments performing at least this number of robotic procedures may provide the minimum operative exposure necessary for young surgeons to progress through the early stages of the learning curve and achieve meaningful gains in performance and self-perceived confidence. In this context, the association between higher institutional volume and higher surgeon confidence observed in our survey likely reflects not only increased hands-on participation but also a more structured and continuous learning environment.

This concept aligns with longstanding evidence linking surgical the quality of training and surgical volume. In fact, centralization of cases has been selected as a required criteria to offer a fellowship in gynecology oncology with the aim to guarantee an adequate surgical volume [17] which has been associated with improved outcomes [18]. In addition, in an interesting survey by European Network of Young Gynaecologic Oncologists (ENYGO), fellows trained in an ESGO accredited centre had a higher chance to perform sentinel lymph node biopsy in cervical cancer [19]. A broader perspective on the volume–outcome relationship is provided by a recent systematic review examining intra-abdominal robotic-assisted surgery. The review reported a positive association between institutional surgical volume and clinical outcomes across gynecologic, urologic, and general surgical procedures, although no consistent annual thresholds were identified in the literature [20]. In a recent Swedish population-based registry study, it was observed that in low-volume hospitals (< 50 procedures/year) and medium-volume hospitals (50–100 procedures/year), laparoscopic hysterectomy was associated with a higher rate of intraoperative complications compared to robotic hysterectomy. Moreover, the study reported conversion rates of 2.2% in low-volume hospitals, 0.9% in medium-volume hospitals, and 0% in high-volume hospitals [21]. These findings are consistent with the notion that surgical volume may impact the quality of outcomes and that this effect is likely more pronounced in the laparoscopic approach given the longer learning curve compared to robotic surgery [22]. Such advantages together with improved ergonomics and enhanced visualization may help explain why robotic surgery was the preferred approach for 46% of respondents in our study.

Structured robotic training, whether fellowship-based or industry-sponsored, was associated with higher confidence in the univariate analysis. It is reasonable to assume that the combination of both training modalities address both clinical and technical aspects, as previously demonstrated in the literature [23]. In this context, surgical simulation has become an essential tool to complement operating room experience, leading to increased confidence and improved performance after structured practice [24] and it has been endorsed by several institutions not only by its proven educational benefits but also by the observed reduction in surgical volumes in training programs [25, 26]. In this regard, significant efforts are underway by SERGS with the aim to develop a European consensus on core components of a curriculum for training and assessment in robot assisted gynecological surgery [27, 28] highlighting the importance of a structured training [23]. Furthermore, there have been collaborative initiatives between the United States and Europe to develop Proficiency-based Progression Train-the-Trainer Courses for Robotic Surgery Training [29] which have proven to be an effective educational framework for gynecologic surgical trainees and robotic teams [30, 31].

Despite 83.9% of departments performing robotic surgery at least once per week, only 37.1% of junior surgeons reported participating in robotic procedures with the same frequency. Limited access likely contributes to the low satisfaction rates observed in this study, with only 17.7% of participants expressing satisfaction with their current exposure. These findings mirror a recent national survey of ACGME-accredited OB/GYN residents in the United States, where 60% of trainees reported minimal hands-on robotic experience by the end of residency [32]. An additional observation in our study was that 9.3% of consultants were practicing in a subspecialty different from their original training. While this might reflect a voluntary shift in professional interests, it may also indicate a disconnect between specialized training pathways and actual job availability, particularly in niche or low-volume fields such as urogynecology or gynecologic oncology. This potential mismatch may contribute to frustration among early-career specialists and merits further investigation.

The relatively small sample size represents a key limitation, contributing to wide confidence intervals, particularly in multivariate analyses. This likely reflects the current limited exposure to robotic surgery among junior European gynecologic surgeons, with senior consultants still performing the majority of procedures. However, this dynamic is expected to shift in the near future, supported by the commitment to training among senior surgeons and the increasing availability of robotic platforms worldwide. We also observed a predominance of respondents with a specific interest in gynecologic oncology, which may reflect greater exposure to robotic surgery within this subspecialty. These findings are consistent with the longstanding tradition of robotic surgery in this field, as further demonstrated in a recent bibliometric study [33]. Among 838 publications examined, 41.1% focused on gynecologic oncology, making it the most prevalent topic. These results call for caution when extrapolating our findings to other areas of interest. Despite these limitations, this study provides preliminary insights into current patterns of exposure and confidence in robotic surgery among young gynecologic surgeons in Europe.

## Conclusions

Gynecologic robotic surgery is increasingly incorporated into surgical practice across Europe, yet access and exposure remain variable among early-career surgeons. In this survey, higher self-perceived confidence in performing robotic surgery was associated with being older than 35 years and with having regular access to robotic operating time. Although these findings must be interpreted with caution, they underscore the potential importance of cumulative experience and consistent operative exposure in developing confidence with robotic procedures. These preliminary results may help inform future training strategies.

## Supplementary Information

Below is the link to the electronic supplementary material.

Supplementary Material 1

## Data Availability

No datasets were generated or analysed during the current study.

## References

[1] Diaz-Arrastia C, Jurnalov C, Gomez G, Townsend C (2002) Laparoscopic hysterectomy using a computer-enhanced surgical robot. Surg Endosc 16(9):1271–1273 10.1007/s00464-002-8523-5 12085153

[2] Glent JCF, Thorgersen EB (2024) Current status and outlook of robotic surgery in the Nordic countries. Scand J Surg 113(1):28–30 10.1177/14574969231211078 37974419

[3] Sinha R, Jain V, Sp S, Saha SC, Sunkavalli C, Kiran L et al (2023) Multi-institutional trends in gynecological robotic surgery in India: a real-world scenario. Cureus. 10.7759/cureus.36564 PMC10122180 37095794

[4] Lycke KD, Kahlert J, Damgaard R, Mogensen O, Hammer A (2021) Trends in hysterectomy incidence rates during 2000–2015 in Denmark: shifting from abdominal to minimally invasive surgical procedures. Clin Epidemiol 13:407–416 10.2147/CLEP.S300394 PMC8180274 34103999

[5] Harvey V, Pfeiffer RM, Landy R, Wentzensen N, Clarke MA (2022) Trends and predictors of hysterectomy prevalence among women in the United States. Am J Obstet Gynecol 227(4):611.e1-611.e12 10.1016/j.ajog.2022.06.028 PMC9529796 35764133

[6] Galvin D, Reilly BO, Greene R, Donoghue KO, Sullivan OO (2023) A national survey of surgical training in gynaecology: 2014–2021. Eur J Obstet Gynecol Reprod Biol 288:135–41 10.1016/j.ejogrb.2023.07.013 37517105

[7] McCormack L, Nesbitt-Hawes E, Deans R, Alonso A, Lim C, Li F et al (2022) A review of gynaecological surgical practices for trainees and certified specialists in Australia by volume using MBS and AIHW databases. Aust N Z J Obstet Gynaecol 62(4):574–80 10.1111/ajo.13523 PMC9542106 35474508

[8] Ruiz MP, Chen L, Hou JY, Tergas AI, Clair CMS, Ananth CV et al (2018) Outcomes of hysterectomy performed by very low-volume surgeons. Obstet Gynecol 131(6):981–90 10.1097/AOG.0000000000002597 PMC5970072 29742669

[9] Klebanoff JS, Marfori CQ, Vargas MV, Amdur RL, Wu CZ, Moawad GN (2020) Ob/Gyn resident self-perceived preparedness for minimally invasive surgery. BMC Med Educ 20(1):1–8 10.1186/s12909-020-02090-9 PMC7275515 32503585

[10] Meyer R, Schneyer RJ, Hamilton KM, Levin G, Truong MD, Siedhoff MT et al (2025) The impact of minimally invasive gynecologic surgery subspecialty training on outcomes of benign laparoscopic hysterectomy: a retrospective cohort study. J Minim Invasive Gynecol 32(2):143–50 10.1016/j.jmig.2024.09.012 39305984

[11] Pfeuti CK, Makai G (2024) Gynecologic surgical subspecialty training decreases surgical complications in benign minimally invasive hysterectomy. J Minim Invasive Gynecol 31(3):250–257 10.1016/j.jmig.2023.12.011 38151094

[12] Douglass KM, Crawford KD, Yazdany T (2024) The impact of fellowship affiliation on urogynecology training in obstetrics and gynecology residency. J Minim Invasive Gynecol 31(12):1004–1010 10.1016/j.jmig.2024.08.004 39147017

[13] Lee YJ, Lee DE, Oh HR, Ha HI, Lim MC (2022) Learning curve analysis of multiport robot-assisted hysterectomy. Arch Gynecol Obstet 306(5):1555–1561 10.1007/s00404-022-06655-5 35767099

[14] Yotsumoto F, Sanui A, Ito T, Miyahara D, Yoshikawa K, Shigekawa K (2022) Cumulative summation analysis of learning curve for robotic-assisted hysterectomy in patients with gynecologic tumors. Anticancer Res 42(8):4111–4117 10.21873/anticanres.15909 35896236

[15] Akazawa M, Hashimoto K, Lee SL, Liu WM (2021) Learning curve of robotic-assisted hysterectomy with pelvic lymphadenectomy for early stage endometrial cancer: analysis of 81 cases. Anticancer Res 41(8):4173–4178 10.21873/anticanres.15222 34281890

[16] Vizza R, Garzon S, Corrado G, Bruno V, Baiocco E, Giannini A (2025) Evaluating the learning curve in robot-assisted laparoscopic total hysterectomy: single-port versus multi-port Da Vinci platforms. J Robot Surg 19(1):733 10.1007/s11701-025-02928-8 PMC12579705 41175288

[17] ESGO accreditation & re-accreditation of European training centres in gynaecological oncology. Available: https://www.esgo.org/media/2021/03/1-SOPs-Training-centres-accreditation-reaccreditation-2021.pdf

[18] Luyckx M, Jouret M, Sawadogo K, Waterkeyn M, Grandjean F, Van Gossum JP et al (2023) Centralizing surgery for ovarian cancer in a non-centralizing country (Belgium): the UNGO (UCLouvain network of gynaecological Oncology) experience. Int J Gynecol Cancer 34(1):106–112 10.1136/ijgc-2023-004401 PMC10850666 37844964

[19] Bizzarri N, Pletnev A, Razumova Z, Zalewski K, Theofanakis C, Selcuk I et al (2022) Quality of training in cervical cancer radical surgery: a survey from the European network of young gynaecologic oncologists (ENYGO). Int J Gynecol Cancer 32(4):494–501 10.1136/ijgc-2021-002812 34992130

[20] Day EK, Galbraith NJ, Ward HJT, Roxburgh CS (2023) Volume-outcome relationship in intra-abdominal robotic-assisted surgery: a systematic review. J Robot Surg 17(3):811–826 10.1007/s11701-022-01461-2 36315379

[21] Brunes M, Forsgren C, Warnqvist A, Ek M, Johannesson U (2021) Assessment of surgeon and hospital volume for robot-assisted and laparoscopic benign hysterectomy in Sweden. Acta Obstet Gynecol Scand 100(9):1730–1739 10.1111/aogs.14166 33895985

[22] Leijte E, de Blaauw I, Van Workum F, Rosman C, Botden S (2020) Robot assisted versus laparoscopic suturing learning curve in a simulated setting. Surg Endosc 34(8):3679–89 10.1007/s00464-019-07263-2 PMC7326898 31754849

[23] Falconer H (2021) Evaluating robotic surgical courses: structured training matters. J Gynecol Oncol 32(2):1–2 10.3802/jgo.2021.32.e39 PMC7930443 33650340

[24] Boitano TKL, Smith HJ, Cohen JG, Rossi EC, Kim KH (2021) Implementation and evaluation of a novel subspecialty society fellows robotic surgical course: the Sgo minimally invasive academy surgical curriculum. J Gynecol Oncol 32(2):1–7 10.3802/jgo.2021.32.e26 PMC7930459 33470068

[25] Hoffman MS, Xiong Y, Apte S, Roberts W, Wenham RM (2019) Twenty-year surgical trends in a gynecologic oncology fellowship training program: implications for practice. Gynecol Oncol 155(2):359–364 10.1016/j.ygyno.2019.09.013 31575391

[26] Stickrath E, Alston M (2017) A novel abdominal hysterectomy simulator and its impact on obstetrics and gynecology residents’ surgical confidence. MedEdPORTAL J Teach Learn Resour 13:10636 10.15766/mep_2374-8265.10636 PMC6338252 30800837

[27] Rusch P, Ind T, Kimmig R, Maggioni A, Ponce J, Zanagnolo V et al (2019) Recommendations for a standardised educational program in robot assisted gynaecological surgery: consensus from the society of European robotic gynaecological surgery (SERGS). Facts Views Vis ObGyn 11(1):29–41 PMC6822956 31695855

[28] Rusch P, Kimmig R, Lecuru F, Persson J, Ponce J, Degueldre M et al (2018) The Society of European Robotic Gynaecological Surgery (SERGS) pilot curriculum for robot assisted gynecological surgery. Arch Gynecol Obstet 297(2):415–20 10.1007/s00404-017-4612-5 PMC5778155 29236172

[29] Collins JW, Levy J, Stefanidis D, Gallagher A, Coleman M, Cecil T et al (2019) Utilising the Delphi process to develop a Proficiency-based progression Train-the-trainer course for robotic surgery training. Eur Urol 75(5):775–785 10.1016/j.eururo.2018.12.044 30665812

[30] Azadi S, Green IC, Arnold A, Truong M, Potts J, Martino MA (2021) Robotic surgery: the impact of simulation and other innovative platforms on performance and training. J Minim Invasive Gynecol 28(3):490–495 10.1016/j.jmig.2020.12.001 33310145

[31] Rivero-Moreno Y, Echevarria S, Vidal-Valderrama C, Stefano-Pianetti L, Cordova-Guilarte J, Navarro-Gonzalez J et al (2023) Robotic surgery: A comprehensive review of the literature and current trends. Cureus. Jul 24;15(7):e42370 10.7759/cureus.42370 PMC10445506 37621804

[32] Adkoli A, Eng S, Stephenson R (2024) Need for formalized robotic training and curriculum in obstetrics and gynecology residency: an examination of current resident outlooks and perspectives. J Robot Surg 18(1):1–7 10.1007/s11701-024-01985-9 PMC11108913 38771400

[33] Levin G, Siedhoff M, Wright KN, Truong MD, Hamilton K, Brezinov Y et al (2023) Robotic surgery in obstetrics and gynecology: a bibliometric study. J Robot Surg 17(5):2387–97 10.1007/s11701-023-01672-1 PMC10492767 37429970

