# Correction: Evaluation of robotic exposure among gynecological surgeons: results of survey from the young European advocates of robotic surgery (YEARS)

**DOI:** 10.1007/s11701-026-03368-8

**Published:** 2026-05-02

**Authors:** Sergi Fernandez-Gonzalez, D. El-Hamamsy, E. Karatrasoglou, A. Amirthanayagam, A. Muñoz Solano, C. Chollet, D. Galvin, M. Manpreet Kaur, C. Uwins

**Affiliations:** 1 Department of Gynaecology, Bellvitge University Hospital (IDIBELL), L’Hospitalet de Llobregat, Spain; 2 Royal London Hospital, London, UK; 3 Euroclinic Group of Hospitals Athens, Alexandra University Hospital, Athens, Greece; 4 Leicester Cancer Research Centre, University of Leicester, Leicester, UK; 5 Hospital Universitario Marqués de Valdecilla, Santander, Spain; 6 Centre Hospitalier Universitaire de Toulouse, Toulouse, France; 7 Surgical Oncology, Institut Universitaire du Cancer Toulouse Oncopole, Toulouse, France; 8 Cork University Maternity Hospital, Cork, Ireland; 9 Chelsea and Westminster Hospital London, London, UK; 10 University Hospital Southampton NHS Foundation Trust, Southampton, UK

**Correction: Journal of Robotic Surgery (2026) 20:241**

10.1007/s11701-026-03153-7

In the original version of the article, the author name was incorrectly given as C. COLLET but should have been C. CHOLLET.

The original version of the article has been corrected.

