# Supplementary material for: Evaluation of robotic exposure among gynecological surgeons: results of survey from the young European advocates of robotic surgery (YEARS)
Source: J Robot Surg. 2026 Feb 11;20(1):241. doi: 10.1007/s11701-026-03153-7 (PMC12891101; doi:10.1007/s11701-026-03153-7)
Supplement: Supplementary file 1 — Supplementary Material 1 [file 11701_2026_3153_MOESM1_ESM.docx]

SUPPLEMENTARY MATERIAL

Document S1. Survey.

**Evaluation of Robotic Exposure Among Gynecological Surgeons**

**Title:** Evaluation of robotic exposure among gynecological surgeons: a survey from the Young European Advocates of Robotic Surgery (YEARS)

**Introduction:** The main objective of this survey is to evaluate the surgical experience of young surgeons in the field of gynecology. It is intended for **all OB/GYN residents** up to **gynecologists who finished less than 4 years ago** their fellowship/training program.

**Inclusion Criteria:**

- Trainee/resident enrolled in a national training programme for obstetrics and gynecology
- Junior doctor working in a non-training post
- Gynecology Clinical Fellows
- Recent graduate (within 3 years) of a fellowship/sub-specialty training programme

**1. How old are you?**

- 24 - 29
- 30 - 34
- 35 - 39
- 40 - 44
- ≥45

**2. What is your gender?**

- Female
- Male
- Other (specify)

**3. In what country do you work?**

**4. Where do you work?**

- Tertiary University Hospital
- Private Hospital
- Tertiary center non-university
- Primary or secondary center

**5. Type your hospital’s name:**

**6. What is your gynecological subspecialty or area of interest?**

- Urogynecology
- Benign gynecology (abnormal uterine bleeding, fibroids, endometriosis, etc.)
- Oncological Gynecology
- Other (please specify)

**7. Does your country have a structured fellowship program for your area of interest?**

- Yes
- No

**8. What is your level of experience within your current role?**

- Currently enrolled in a specialty training program/residency in OB/GYN
- In the first half of a post-graduate fellowship/training program
- In the second half of a post-graduate fellowship/training program
- In a non-training position
- Finished a training program and working as a consultant
- I haven’t done any training program

**9. Are (or were) you enrolled in a sub-specialty structured training program?**

- My training program is (or was) a structured fellowship
- My training program is (or was) a non-structured fellowship
- I am (or was) not enrolled in any training program
- Other (please specify)

**10. If you have finished your post-graduate/subspecialty training, are you working in your area of interest?**

- Yes, I am working according to my completed training program
- No, I am working in other competencies
- Other (please specify)

**11. Is there a surgical robotic device at your workplace?**

- Yes
- No

**12. When did your department start to perform robotic surgery?** (Type the year)

**13. What kind of training have you had regarding ROBOTIC SURGERY?**

- None
- Industry course
- National or international society course
- Local hospital course
- Fellowship or subspecialty training
- Other (please specify)

**14. Which robotic device do you use?** (More than one answer possible)

- Da Vinci Si (Intuitive)
- Da Vinci X (Intuitive)
- Da Vinci Xi (Intuitive)
- Hugo (Medtronic)
- Versius (CMR)
- Hominis (Surgical Science)
- Other (please specify)

**15. How often does your department perform robotic surgery?**

- 1 day per week
- 1 day per week
- 1 day every 2 weeks
- < 1 day every 2 weeks
- Never

**16. Is your department currently involved in robotics research?**

- No and it has never been
- No but it was in the past
- Yes, in retrospective studies
- Yes, in prospective studies

**17. How many abdominal/pelvic surgeries (average) does your department perform per year by any approach?**

**18. How many surgeries (average) does your department perform per year by robotic approach?**

**19. Do you operate as a main surgeon by robotic approach?**

- 1 day per week
- 1 day every 2 weeks
- < 1 day every 2 weeks
- <1 day per month
- No

**20. Would you like to operate more often by a robotic approach?**

- No, I am happy with my frequency as main surgeon
- Yes, I would like to operate as a main surgeon more often
- Yes, I would like to initiate performing surgeries as a main surgeon

**21. Which is the main activity performed by robotics in your department?** (More than one is possible)

- Benign surgery (endometriosis, fibroids, fertility surgery, etc.)
- Gynecological Oncology
- Urogynecology
- Breast surgery

**22. Which is the main procedure that you perform as a main surgeon by robotics?**

- Benign Hysterectomy
- Adnexectomy
- Endometriosis resection
- Malignant Hysterectomy + lymph node assessment (SLNB or lymphadenectomy)
- Cytoreduction
- Colposacropexy/colposuspension
- Other (please specify)

**23. Regarding the last question, how do you feel more confident to perform as a main surgeon?**

- Open surgery
- Laparoscopic
- Robotic
- I do not have enough experience yet

**24. Do you feel confident in performing a main procedure of your subspecialty?**

- Not yet, I need to assist more cases as first assistant
- Not yet, I need to assist more cases as second assistant
- Yes, assisted by a consultant
- Yes, assisted by fellows/residents with direct consultant supervision
- Yes, without any supervision

**25. What learning tools do you access most frequently about robotics?** (More than one allowed)

- Dedicated robotic on-site courses
- Webinars
- Society websites
- YouTube
- Social Media
- Other (please specify)

**26. Would you like to do a short observership in order to learn more about robotic surgery?**

- Extremely interested
- Very interested
- Somewhat interested
- Not so interested
- Not at all interested

**27. How do you define access to robotic learning tools (didactic training, courses, videos, etc.)?**

- Easy access, there is a lot of information available
- Moderate access, not enough information available
- Difficult access, very limited content available

**28. Please let us know any comments you have:** [Free text box]

Document S2. Checklist for Reporting Results of Internet E-Surveys (CHERRIES)^1^

| ***Checklist Item*** | ***Explanation*** | ***Page Number*** |
| --- | --- | --- |
| Describe survey design | Describe target population, sample frame. Is the sample a convenience sample? (In “open” surveys this is most likely.) | p. 5-6 (Methods: Study design) |
| IRB approval | Mention whether the study has been approved by an IRB. | p. 5 (Methods: Ethics) |
| Informed consent | Describe the informed consent process. Where were the participants told the length of time of the survey, which data were stored and where and for how long, who the investigator was, and the purpose of the study? | p. 5 (Methods: Ethics) |
| Data protection | If any personal information was collected or stored, describe what mechanisms were used to protect unauthorized access. | p. 6 (Methods: Data handling - confidentiality mentioned) |
| Development and testing | State how the survey was developed, including whether the usability and technical functionality of the electronic questionnaire had been tested before fielding the questionnaire. | p. 5 (Methods: Questionnaire design) |
| Open survey versus closed survey | An “open survey” is a survey open for each visitor of a site, while a closed survey is only open to a sample which the investigator knows (password-protected survey). | p. 5-6 (Methods: Setting/Recruitment) |
| Contact mode | Indicate whether or not the initial contact with the potential participants was made on the Internet. (Investigators may also send out questionnaires by mail and allow for Web-based data entry.) | p. 5-6 (Methods: Setting/Recruitment) |
| Advertising the survey | How/where was the survey announced or advertised? Some examples are offline media (newspapers), or online (mailing lists – If yes, which ones?) or banner ads (Where were these banner ads posted and what did they look like?). It is important to know the wording of the announcement as it will heavily influence who chooses to participate. Ideally the survey announcement should be published as an appendix. | p. 5-6 (Methods: Setting/Recruitment) |
| Web/E-mail | State the type of e-survey (eg, one posted on a Web site, or one sent out through e-mail). If it is an e-mail survey, were the responses entered manually into a database, or was there an automatic method for capturing responses? | p. 5-6 (Methods: Platforms) |
| Context | Describe the Web site (for mailing list/newsgroup) in which the survey was posted. What is the Web site about, who is visiting it, what are visitors normally looking for? Discuss to what degree the content of the Web site could pre-select the sample or influence the results. For example, a survey about vaccination on a anti-immunization Web site will have different results from a Web survey conducted on a government Web site | p. 5-6 (Methods: Setting - SERGS/YEARS networks) |
| Mandatory/voluntary | Was it a mandatory survey to be filled in by every visitor who wanted to enter the Web site, or was it a voluntary survey? | p. 5-6 (Methods: Voluntary participation) |
| Incentives | Were any incentives offered (eg, monetary, prizes, or non-monetary incentives such as an offer to provide the survey results)? | No incentives |
| Time/Date | In what timeframe were the data collected? | p. 6 (Methods: Responses accepted Dec 2022–Sep 2023) |
| Randomization of items or questionnaires | To prevent biases items can be randomized or alternated. | No randomization |
| Adaptive questioning | Use adaptive questioning (certain items, or only conditionally displayed based on responses to other items) to reduce number and complexity of the questions. | p. 5 (Methods) |
| Number of Items | What was the number of questionnaire items per page? The number of items is an important factor for the completion rate. | p. 5 (Methods: 28 questions) |
| Number of screens (pages) | Over how many pages was the questionnaire distributed? The number of items is an important factor for the completion rate. | p. 5 (Methods) |
| Completeness check | It is technically possible to do consistency or completeness checks before the questionnaire is submitted. Was this done, and if “yes”, how (usually JAVAScript)? An alternative is to check for completeness after the questionnaire has been submitted (and highlight mandatory items). If this has been done, it should be reported. All items should provide a non-response option such as “not applicable” or “rather not say”, and selection of one response option should be enforced. | p. 5 (Methods) |
| Review step | State whether respondents were able to review and change their answers (eg, through a Back button or a Review step which displays a summary of the responses and asks the respondents if they are correct). | p. 5 (Methods) |
| Unique site visitor | If you provide view rates or participation rates, you need to define how you determined a unique visitor. There are different techniques available, based on IP addresses or cookies or both. | Not participation rates |
| View rate (Ratio of unique survey visitors/unique site visitors) | Requires counting unique visitors to the first page of the survey, divided by the number of unique site visitors (not page views!). It is not unusual to have view rates of less than 0.1 % if the survey is voluntary. | Not counting visitors |
| Participation rate (Ratio of unique visitors who agreed to participate/unique first survey page visitors) | Count the unique number of people who filled in the first survey page (or agreed to participate, for example by checking a checkbox), divided by visitors who visit the first page of the survey (or the informed consents page, if present). This can also be called “recruitment” rate. | Not recruitment rate visitors |
| Completion rate (Ratio of users who finished the survey/users who agreed to participate) | The number of people submitting the last questionnaire page, divided by the number of people who agreed to participate (or submitted the first survey page). This is only relevant if there is a separate “informed consent” page or if the survey goes over several pages. This is a measure for attrition. Note that “completion” can involve leaving questionnaire items blank. This is not a measure for how completely questionnaires were filled in. (If you need a measure for this, use the word “completeness rate”.) | p. 6 (Results: 81 completed surveys; denominator not available) |
| Cookies used | Indicate whether cookies were used to assign a unique user identifier to each client computer. If so, mention the page on which the cookie was set and read, and how long the cookie was valid. Were duplicate entries avoided by preventing users access to the survey twice; or were duplicate database entries having the same user ID eliminated before analysis? In the latter case, which entries were kept for analysis (eg, the first entry or the most recent)? | p. 5 (Methods) |
| IP check | Indicate whether the IP address of the client computer was used to identify potential duplicate entries from the same user. If so, mention the period of time for which no two entries from the same IP address were allowed (eg, 24 hours). Were duplicate entries avoided by preventing users with the same IP address access to the survey twice; or were duplicate database entries having the same IP address within a given period of time eliminated before analysis? If the latter, which entries were kept for analysis (eg, the first entry or the most recent)? | p. 5 (Methods) |
| Log file analysis | Indicate whether other techniques to analyze the log file for identification of multiple entries were used. If so, please describe. | p. 5 (Methods) |
| Registration | In “closed” (non-open) surveys, users need to login first and it is easier to prevent duplicate entries from the same user. Describe how this was done. For example, was the survey never displayed a second time once the user had filled it in, or was the username stored together with the survey results and later eliminated? If the latter, which entries were kept for analysis (eg, the first entry or the most recent)? | Not applicable (open survey) |
| Handling of incomplete questionnaires | Were only completed questionnaires analyzed? Were questionnaires which terminated early (where, for example, users did not go through all questionnaire pages) also analyzed? | p. 6 (Methods: Only completed surveys analyzed) |
| Questionnaires submitted with an atypical timestamp | Some investigators may measure the time people needed to fill in a questionnaire and exclude questionnaires that were submitted too soon. Specify the timeframe that was used as a cut-off point, and describe how this point was determined. | It was not analyzed |
| Statistical correction | Indicate whether any methods such as weighting of items or propensity scores have been used to adjust for the non-representative sample; if so, please describe the methods. | p. 6-7 (Methods: Statistical analysis) |

REFFERENCES

1. Eysenbach G. Improving the quality of Web surveys: the Checklist for Reporting Results of Internet E-Surveys (CHERRIES). J Med Internet Res. 2004 Sep 29;6(3):e34.
